# Eat UP: a precise, science-based, value-aware, translatable diet quality metric for public health nutrition

**DOI:** 10.1017/S1368980026102201

**Published:** 2026-03-02

**Authors:** Beverley O’Hara

**Affiliations:** School of Health, Leeds Beckett University, Leeds, UK

**Keywords:** Diet quality, Nutrition epidemiology, Unrefined plantfoods, Public Health

## Abstract

Current approaches to measurement of diet quality lack precision, do not translate well beyond academia and may have unintended consequences. A novel metric ‘unrefined plantfoods’ (UP) is proposed. The UP metric is grounded in established science, precise, easy to measure, consistent with wider nutrition agendas and policies, reduces potential commercial and ideological exploitation of public health nutrition goals, is unbiased, translatable and inclusive. The concept is value-aware and grounded in a harm minimisation approach.

Assessment of diet quality is a fundamental element of nutritional epidemiology, essential for monitoring public health, and also of increasing interest to consumers. There are three approaches – nutrient profiling, food profiling and dietary pattern profiling. Nutrient profiling is a reductionist approach, which ignores overall dietary context. There are currently 167 known nutrient profile models in use and fewer than half of these include information on their validation^(1)^. The widely used NOVA profiling system is based on the extent and purpose of food processing^(2)^. Despite the poorly defined nature of ‘ultra-processed’ foods (UPF) and the conflation of processing methods with assumed health effects^(3)^, their quantification in diets has become a focus in nutrition research, generating a substantial body of flawed literature^(4)^ and reviews based on low-quality evidence^(5)^. Dietary pattern profiling aims to characterise diet or calculate a score (e.g. the Healthy Eating Index^(6)^) based on the level of adherence to a purported ‘healthy’ dietary pattern. This method also has inherent limitations. For example, there is heterogeneity in definitions of the extensively studied ‘Mediterranean’ diet and no universally agreed definition of adherence^(7)^. Furthermore, the nomenclature is problematic in suggesting that dietary components from the Mediterranean region (e.g. olive oil) are uniquely beneficial. In fact, the main benefits of the diet likely stem from the substantially higher consumption of plantfoods among populations in Mediterranean regions with traditional food cultures, compared to populations in industrialised regions. This hypothesis is congruent with the most likely mechanism underlying observed UPF-health associations, namely the displacement of diets centred on whole foods^(2)^.

Robust examination of the relationship between diet quality and health outcomes necessitates well-defined exposure variables; as such, greater standardisation in the reporting of dietary patterns research is required^(7)^. Improved methods will facilitate the development of unequivocal, evidence-informed policy guidance, while avoiding research waste and opportunity costs related to nutrition research funding. To address this need, a new metric to measure diet quality is proposed, alongside a comprehensive rationale. The proposed metric provides measurable, mechanistically plausible criteria for assessing diet quality whilst maintaining practical applicability for diverse populations and food systems.

The term ‘unrefined plantfood’(UP) describes any edible plant that has an intact (unrefined) matrix. Adopting measurement of UP as a diet quality metric has distinct advantages over current approaches. The concept is:

1. **Grounded in established science**

It is well-established that intake of fruit and vegetables, legumes, whole grains and nuts is beneficial for human health^(8)^. UP benefit health by providing essential vitamins and minerals along with phytochemicals and other nutritional ‘dark matter’^(9)^. Their fibre content supports digestive health, promotes beneficial gut bacteria and slows nutrient absorption; their intact cellular structures can limit bioaccesibility and bioavailability of nutrients^(10)^, reducing glycaemic responses, and they typically have low energy density. The UP metric allows for an assessment of diet quality based on the relative proportion of foods in a dietary pattern that are associated with a reduction in risk of poor health outcomes.

1. **Precise, easy to understand and translatable**

UP are easy to classify. Any edible plant that has not undergone industrial milling or grinding is an UP. Previously, the term ‘wholefoods’ has been used to describe these foods; however, this term is imprecise. One issue is that there is no single definition of whole grain products^(11)^, leading to a fragmented landscape where definitions and categorisation vary by country. Use of the term ‘unrefined plantfoods’ avoids inconsistencies and confusion over what is, or is not, a ‘wholefood’ or ‘whole grain’^(12)^ and is therefore translatable across contexts. For example, it could be applied in countries where healthy sales targets are set for retailers to monitor the shift towards healthy sustainable diets^(13)^.

Terminology used in food classification systems will inevitably extend beyond academic discourse into broader public, media and policy contexts and should therefore be accessible and understandable to a wide range of people in diverse circumstances. It should also correspond with established knowledge and intuition while bolstering consumers’ confidence in their ability to identify healthy food. The UPF paradigm is deficient in this sense; consumers have high levels of awareness but low levels of understanding of the concept^(14)^, and attempts to refine the classification system – for example, the introduction of a ‘superultra-processed’ category – may lead to increased confusion^(15)^.

1. **Easy to measure, analyse, interpret and report**

The UP metric has significant utility in nutrition epidemiology. Intake of UP can be computed from quantitative or semi-quantitative dietary intake and do not require food composition data. The simplicity of the metric allows for comparable and reproducible studies^(7)^ and has relative validity in diverse population groups as well as scope for the development of rapid screening tools which could be used at very low cost in a range of contexts. Retrospective analyses of existing data on diet and health outcomes using UP as a diet quality metric may have greater validity than many existing studies. Such analyses would allow for development of a validated indicator threshold.

1. **Positively framed and consistent with existing nutrition-health agendas**

The UP paradigm provides a basis for the communication of clear health messages which do not oversimplify the very complex factors that interact to constitute a ‘healthy’ diet. Advice to eat more UP is simple, actionable, positive^(16)^ and consistent with existing nutrition messages (e.g. the Planetary Health Diet)^(17)^ and aligns with policy priorities for CVD, diabetes and obesity^(18)^. Evidence suggests that policies encouraging consumption of dietary components currently consumed below recommended levels may yield greater benefits than those focused exclusively on nutrients to limit^(19)^. UP-based dietary advice is pragmatic, theoretically achievable within existing food systems and does not require large-scale food system transformation.

1. **Informed by principles of harm minimisation**

The concepts and terminology employed within nutrition science hold considerable potential for application across broader contexts and should therefore align not only with the values of nutrition science^(20)^ but also with public health practice. The potential for direct, psychological, equity, group, social and opportunity cost harms^(21)^ must be considered. Oversimplification and use of alarmist messages relating to public health nutrition is likely to have the longer-term effect of eroding trust in nutrition science^(22)^, with resultant harm to society. To nurture trust, nutrition discourse should be positive^(16)^, measured and prioritise the groups and communities most vulnerable to diet-related harm^(23)^.

Reducing health inequities is a key tenet of harm minimisation. Being stigmatising to low-income groups, simplistic anti-processing narratives may lead to other downstream harms. For example, taxation of staple foods may disproportionately impact socioeconomically disadvantaged communities^(24)^. Other unintended social harms include anxiety and social stress about foods that are perceived as potentially harmful^(25)^ and the misrepresentation of processing and ‘the food industry’ as inherently problematic, leading to the avoidance of processed foods. In extreme cases, such beliefs and behaviours may contribute to the development of eating difficulties (e.g. Orthorexia Nervosa) in vulnerable individuals^(26)^. Food science and technology are central to creating safe, affordable food products that support modern lifestyles and will play a key role in the protein transition. Activities that undermine consumer confidence in processed food products (e.g. alternative proteins) should be avoided^(27)^.

1. **Independent of ideology**

To measure exposures and elucidate clear causal pathways in nutrition epidemiology, foods should be categorised based on the properties of the food itself rather than its means or purpose of production, distribution or marketing. The UPF concept, whilst valuable in highlighting concerns about global food systems, fails to meet these requirements; however, its widespread adoption demonstrates that the concept resonates with public concerns – consumers value clarity and certainty in dietary guidance. Nevertheless, recognition of this appeal does not negate the need for a more robust, scientifically grounded, equitable and culturally translatable alternative^(28)^.

The UP framework satisfies this requirement. It is ideologically neutral and may therefore be integrated into legislation with broader acceptance than systems based on level of processing^(29)^, which imply that processing *per se* is problematic even though this is not the case.

1. **Unhackable by commercial actors**

Commercial pressures for continuous innovation mean that the food industry capitalises on emerging trends in nutrition science to develop and market new products. On one hand, such innovation can lead to the production of industrialised food products with improved nutritional profiles. Conversely, there is abundant evidence of the cynical appropriation of advances in nutrition science (e.g. the role of vitamins in health; the benefits of consuming whole grains) that have led to misleading health claims on products of low nutritional quality (e.g. fortified but highly sweetened ready-to-eat breakfast cereals). Commercial actors can respond rapidly to monetise consumer awareness of advances in nutrition. For example, recent increased consumer interest in less processed foods has led to the creation of novel milling techniques that result in higher proportions of intact cells, so-called ‘nutritional capsules’ (Xiong *et al.*, 2022)^(10)^ as well as certification schemes (e.g. The Non-UPF Verified Standard)^(30)^ and an UPF identification app^(31)^. Health messaging based on the concept of UP would reduce the scope for commercial exploitation of public health nutrition goals.

1. **Unbiased**

Whilst it is widely understood that nutrition science can be vulnerable to distortion and bias from commercial interests^(32)^, distortion and bias from media influence, non-financial conflicts of interest and advocacy are less acknowledged^(33)^. Strong allegiance to specific hypotheses^(34)^ regardless of a lack of consensus on concepts, methods and terminology (e.g. some research on UPF^(35)^), white hat bias and investigator bias have the potential to further erode trust in nutrition science^(36)^. The UP paradigm is explicitly grounded in the Mertonian values of universalism, disinterestedness and organised scepticism^(37)^ and is free from bias.

1. **Broadly inclusive**

Concepts used in public health nutrition should be sensitive to equity effects and should not exacerbate existing inequalities by benefitting privileged groups; when used uncritically, health policy language can reinforce power imbalances^(38)^. The way that issues in nutrition are framed, defined and communicated must be culturally and linguistically responsive to the needs of different communities^(38)^. Use of the term ‘unrefined plantfoods’ supports a move away from language that may be classed, privileged (e.g. ‘Real’ food^(39)^), stigmatising^(40)^ (e.g. ‘processed’ food as socially devalued) and moralising^(41)^. The concept fits with wider cultural models of a healthy diet, acknowledging that affordability, access, traditions and cultures vary by region and country, and therefore supports the development of nutrition guidance and policy that promotes equity.

1. **Acknowledges the complexity of food and nutrition systems**

Public health messaging around food and health must acknowledge the complexity of food systems and eating behaviours and not rely on misleading oversimplifications of what ‘good’ and ‘bad’ foods are. Improving diet quality in industrialised regions is a complex and multifaceted issue. Solving this problem will require a transdisciplinary approach grounded in the ecological nutrition paradigm^(42)^ and is beyond the influence of individual consumers. As such, nutrition messaging should emphasise individual agency and empower consumers with clear, actionable information over disseminating approaches that stigmatise entire food categories. The UP paradigm is compatible with this strategy.

## Limitations

In common with other attempts to create a metric to characterise what a ‘good diet’ is, the UP paradigm has inherent limitations. Reducing complex dietary patterns to a few dimensions is a reductionist approach and overlooks potentially ‘zillions’ of variables that may have putative causal effects^(34)^.

The UP metric is centred around dietary components that are known to be associated with reduced risk of noncommunicable diseases. As such, the metric is not a comprehensive measure of overall diet quality, but one aspect of it. Nevertheless, it is likely to be a robust marker of a healthy dietary pattern and can be used in combination with other indices (e.g. proportion of high fat, salt and sugar foods in the diet).

Researchers utilising the UP metric may have decisions to make about individual foods. For example, intake of potatoes and other ‘starchy staples’ is often not classed as vegetable intake in nutrition research; one option would be to report total grams of UP in the diet and grams of potatoes or equivalent separately. Data could also be further stratified by cooking method if required.

## Conclusion

The credibility of nutrition science depends upon the development of precise, evidence-based, ideologically neutral and globally coherent systems for characterising dietary exposures. At the same time, clear dissemination of nutrition science that distinguishes evidence-based information from advocacy and opinion is essential to inform effective policy and interventions. Public health nutrition must proceed with humility, caution and a keen awareness of potential unintended consequences. Only through such an approach can the field develop dietary guidelines and inform policies^(43)^ that are simultaneously scientifically credible, socially equitable, epistemically trustworthy^(44)^ and effective in improving population health.

## Positionality statement

Despite the author’s personal concerns about corporate power and the commercial determinants of health, the author acknowledges that the proposed diet quality metric prioritises biological mechanisms over socio-political concerns about industrialisation and global food systems. This reflects the value that scientific rigour should take precedence over advocacy.
